# Protein contact order prediction from primary sequences

**DOI:** 10.1186/1471-2105-9-255

**Published:** 2008-05-30

**Authors:** Yi Shi, Jianjun Zhou, David Arndt, David S Wishart, Guohui Lin

**Affiliations:** 1 Department of Computing Science, University of Alberta. Edmonton, Alberta, T6G 2E8, Canada; 2 Department of Biological Sciences, University of Alberta. Edmonton, Alberta, T6G 2E9, Canada

## Abstract

**Background:**

Contact order is a topological descriptor that has been shown to be correlated with several interesting protein properties such as protein folding rates and protein transition state placements. Contact order has also been used to select for viable protein folds from *ab initio *protein structure prediction programs. For proteins of known three-dimensional structure, their contact order can be calculated directly. However, for proteins with unknown three-dimensional structure, there is no effective prediction method currently available.

**Results:**

In this paper, we propose several simple yet very effective methods to predict contact order from the amino acid sequence only. One set of methods is based on a weighted linear combination of predicted secondary structure content and amino acid composition. Depending on the number of components used in these equations it is possible to achieve a correlation coefficient of 0.857–0.870 between the observed and predicted contact order. A second method, based on sequence similarity to known three-dimensional structures, is able to achieve a correlation coefficient of 0.977. We have also developed a much more robust implementation for calculating contact order directly from PDB coordinates that works for > 99% PDB files. All of these contact order predictors and calculators have been implemented as a web server (see Availability and requirements section for URL).

**Conclusion:**

Protein contact order can be effectively predicted from the primary sequence, at the absence of three-dimensional structure. Three factors, percentage of residues in alpha helices, percentage of residues in beta strands, and sequence length, appear to be strongly correlated with the absolute contact order.

## Background

Considerable computational and experimental efforts over the past three decades have been devoted to learning about or predicting how proteins fold. Experimentally, insights into protein folding mechanisms can be gained by measuring bulk properties such as protein folding rates [1,2], free energies of folding [3] or hydrogen exchange rates [4] and correlating them with molecular properties such as secondary structure [5], molecular topology [6] and solvent accessibility [7]. One of the more remarkable observations to emerge over the past decade is that protein folding rates vary over many orders of magnitude, from microseconds [2] to hours [1]. These experimental observations, in combination with theoretical studies, have led to a general agreement that protein folding mechanisms and folding landscapes are largely determined by the topology of the native protein and are relatively insensitive either to the details of the inter-atomic interactions [6,8-11] or to the length of the protein [6].

To better quantify the topology and the stability of protein native states, the concept of *contact order *(CO) was proposed in 1998 [6]. Contact order is essentially a measure of non-adjacent amino acid proximity within a folded protein. More specifically, two distinct amino acid residues in a protein are said to form a contact when there is a pair of heavy atoms (C, O, S or N), one from each residue, whose physical (euclidean) distance is within 6 Å [11]. The absolute contact order, denoted as Abs_CO, of a protein is defined as the average number of residues separating the contacts inside the protein (where two sequentially adjacent residues are separated by one residue). The relative contact order, or simply the contact order, is denoted as CO. Essentially, CO measures the average sequence separation between contacting residues in the native state of a protein normalized by the protein length, and intuitively, when the portion of interacting atoms which are far away in the protein sequence grows, CO increases.

Both positive and negative correlations have been found between CO and several bulk protein properties such as protein folding rate and transition state placements [6,11-16]. For example, previous experimental results have shown that the logarithm of a protein's folding rate is linearly correlated with CO of the protein in its native state [11]. A similar but inverse correlation between CO and the protein folding transition state placements has also been observed [6]. Early studies have suggested that Abs_CO exhibited a weaker correlation with two-state protein folding kinetics than CO does [6,9]. More recently, Ivankov *et al. *[11] showed that Abs_CO is a more appropriate parameter to predict the folding rate of proteins as it actually spans a wider range of folding state kinetics (i.e., two-state, multi-state, and short peptides) [11]. Consequently, some of the more promising applications of CO prediction or calculation lie in the prediction of protein folding rates, folding transition state placements, and other folding properties.

In addition to its application in predicting protein folding kinetic properties, contact order has also been shown to have some utility in *ab initio *protein structure prediction [10]. In particular, it has been observed that during the candidate structure generation stage in *ab initio *structure prediction programs, decoys with higher topological complexity are more likely to be under-sampled, especially among larger proteins. Normalizing the CO distribution of candidate structures has been shown to alleviate such a bias, and, as a result, better protein structure predictions were generally achieved [10]. In fact, contact order filtering is now an integral part of the Rosetta protein structure prediction package [17].

In this study, we adopted the CO definition in which two distinct amino acid residues in a protein form a contact when there is a pair of heavy atoms (C, O, S or N), one from each residue, whose physical (euclidean) distance is within 6 Å [6,11]. We note that in the literature, there are several different definitions of CO. For instances, Bonneau *et al. *suggested that two residues form a contacting pair if and only if they are sequentially at least 3 residues away from each other and their *β*-carbons are within 8 Å [10]. Yuan studied different distance thresholds (6 Å, 8 Å, 10 Å, 12 Å, 14 Å) in Plaxco *et al.*'s definition and concluded that they did not significantly affect the prediction accuracy [18]. It has also been suggested that sequentially adjacent residues should not be considered to be a contact in Plaxco *et al.*'s definition. Note that although these variants use different parameters in defining a contact, the underlying ideas of using CO to quantify the topology of a protein's native state tertiary structure are similar. In the literature, there are also several well-studied concepts related to CO such as residue contact order [19-22], contact number [18,23,24], and residue contact number [25-28]. These quantities are largely used to characterize protein native structure, but unlike contact order, they are not directly correlated to some global protein properties such as protein folding rate and folding transition state placements. While some researchers [14,15] have tried to predict protein folding rate from the amino acid sequence directly, typically they only tested their methods on very small data-sets and the results were subject to the overfitting problem [29].

For proteins with solved three-dimensional structures, their COs can be calculated exactly using the equations given below (in Methods), according to the definition given by Plaxco *et al. *[6,11]. In fact, a web server (albeit with limited functionality) has been developed that calculates contact order when given an appropriately formatted PDB coordinate file [30]. However, to the best of our knowledge, there is no CO prediction method available when the three-dimensional structure of the target protein is unknown. Given that only a tiny fraction of protein 3D structures are known and given the utility of contact order in the understanding and prediction of protein folding rates and protein folds, we decided to tackle the problem of predicting CO for proteins with unknown three-dimensional structures (i.e., predicting CO using only the amino acid sequence as input). In addressing this problem we wanted to develop a method that could accurately predict or robustly calculate contact order regardless of whether the 3D structure was known or not. Therefore, three scenarios are possible: 1) the input sequence exactly matches a known 3D structure; 2) the input sequence is homologous (> 20% sequence identity, computed as the number of identical residues divided by the query sequence length) to a known 3D structure and 3) the input sequence does not match any known structure. As described below, we have succeeded in developing a combination of methods that is capable of predicting CO with a correlation between the observed and predicted values ranging from 0.857 (for scenario 3) to 0.977 (for scenario 2) to 1.000 (for scenario 1). Details regarding the implementation, testing and performance of these methods are given below.

## Methods

### Contact order calculation

Given a protein primary sequence, let us use *L *to denote its length, i.e., the number of amino acid residues in the sequence. The *i*-th residue is denoted as *a*_*i*_. For two distinct residues *a*_*i *_and *a*_*j*_, if there are two non-hydrogen atoms, one from each residue, within 6 Å, then *a*_*i *_and *a*_*j *_form a contact. *L*_*ij *_= |*i *- *j*| denotes the number of residues separating this contact. Assuming there are in total *N *contacts in the protein, the Abs_CO of this protein is defined as

(1) $$\text{Abs\_CO}=\frac{1}{N}\sum_{\left(a_{i},a_{j}\right)}L_{ij},$$

where the summation goes over all contacting pairs (*a*_*i*_, *a*_*j*_) in the protein [6,11]. The relative CO, or simply CO, is defined as

(2) $$\text{CO}=\frac{1}{L}\times \text{Abs\_CO}.$$

Because the relative CO is defined as Abs_CO normalized over protein length, exactly the same prediction accuracy can be achieved for Abs_CO as for CO. In this study, we focus on calculating and predicting Abs_CO, from which the corresponding CO can be trivially calculated. We implemented a contact order calculator that determines the Abs_CO value from the PDB coordinates of an input protein using the methods described above. The program was tested and validated against a large number of files for which the Abs_CO values had been previously published.

### Prediction by homology

Many protein properties, including tertiary structure, secondary structure and solvent accessibility can be predicted via homology [31]. In other words, the properties of a query sequence can be predicted by directly transferring the properties or features of a homologous protein to the query protein. Since CO is a property that is a function of structure, we hypothesized that the calculated CO of known 3D structures could be used to predict the CO of homologous proteins. In implementing this approach we calculated the Abs_CO (using the method described in the last "Contact order calculation" section) for 16, 499 non-redundant proteins obtained from the PDB. These proteins were selected using the PDB culling/filtering service called PISCES [32]. Structures were initially selected using a 95% identity sequence-redundancy cutoff and a requirement for better than 3 Å resolution (for X-ray structures). Structures were further processed by removing disordered structures (secondary structure content < 10%) as well as all membrane proteins (membrane beta barrel and transmembrane helix proteins). The resulting CO database consisted of 16, 499 sequences in FASTA format with the Abs_CO value listed in the sequence name header. A local copy of BLAST [33] was installed which used this FASTA-formatted CO database as the search database. For a hit to be considered to be significant the query sequence must exhibit more than 20% sequence identity (computed as the number of identical residues divided by the query sequence length) to a protein in the CO database and the query sequence must be ± 40% of the length of the matching homologue. If these two criteria are met, then the contact order is transferred to the query protein. If any of these criteria is not met, then the contact order is predicted using the method described in the next "Prediction by regression" section. Tests through 5-fold cross validation on the CO database were performed using a variety of sequence identity cutoffs and sequence-length thresholds to assess their influence on both the accuracy and the coverage (coverage refers to the percentage of query sequences that could be predicted by this homology-based method). Overall, the 20% sequence identity cutoff and the 40% length threshold provided the best accuracy-to-coverage tradeoff.

### Prediction by regression

In order to deal with the situation where no homologue can be found to predict the CO value (the last "Prediction by homology" section) we developed and tested a regression-based approach that permits accurate prediction of CO for any water-soluble protein. Let *p*(*α*) denote the percentage of residues in alpha-helices and *p*(*β*) denote the percentage of residues in beta strands in the protein. We observed that Abs_CO correlates well with a linear combination of *p*(*α*), *p*(*β*), and the protein length *L*. Given this observation we decided to use linear regression to optimize the correlation between Abs_CO and the protein primary and secondary structures, as follows:

(3) Abs_CO = *χ*_1 _· *p*(*α*) + *χ*_2 _· *p*(*β*) + *χ*_3 _· *L *+ *c*,

where *χ*_*i*_, *i *= 1, 2, 3, are the coefficients of the three factors *p*(*α*), *p*(*β*), and *L*, and *c *is a constant value in the linear regression. Note that for proteins with unknown three-dimensional structure, their secondary structures are also unknown. Therefore, as part of the linear regression process as specified in Formula (3), we predict their secondary structure content using Proteus [31]. Proteus is a secondary structure predictor that uses sequence alignment to achieve highly accurate predictions (Q3 accuracy score of 81.3% or greater), where homologs are identified using an E-value of < 0.01 and the secondary structures derived from VADAR [34] and the PPT-Database [35].

Using a large dataset of 933 high resolution three-dimensional protein structures (see Results section), the parameters in Formula (3) localize at *χ*_1 _= -6.8968, *χ*_2 _= 7.6216, *χ*_3 _= 0.0612, and *c *= 8.0397.

Subsequently, given any query protein, we may use Proteus again to predict *p*(*α*) and *p*(*β*) values, and then report its Abs_CO as

(4) Abs_CO = -6.8968*p*(*α*) + 7.6216*p*(*β*) + 0.0612*L *+ 8.0397.

In the Results section, we will demonstrate the effectiveness of this stunningly simple prediction method.

In addition to this 3-factor CO predictor (Formula (4), and denoted as F3-LR), which has been implemented on our web server, we also developed other linear equations that considered more factors that might be strongly correlated to Abs_CO. For example, we added four other factors to F3-LR to create a 7-factor linear regression formula. These four factors are 1) the number of beta hairpins (two adjacent beta-strand segments form a hairpin if they are separated by 2 to 5 residues), 2) the number of distant beta strands (two adjacent beta-strand segments are considered "distant" if they are separated by at least 5 residues), 3) the number of Cysteine residues (C), and 4) the number of hydrophobic amino acid residues (V, I, L, M, F, W, C). Among these four factors, the latter two are obtained from the primary sequence, while the former two are extracted from the secondary structures predicted using Proteus. This method is denoted as F7-LR. The third method known as F27-LR considers 27 factors. These 27 factors include the first 5 factors in the F7-LR method, (the other two factors in the F7-LR method, the number of Cysteine residues and the number of hydrophobic amino acid residues, are replaced by) 19 amino acid frequencies of the 20 ones in the target protein, and 3 hydrophobicity frequencies defined as follows. For each amino acid type, its frequency in the target protein is defined as the number of occurrences divided by *L*, the length of the protein. Since the sum of all 20 such frequencies is 1, only 19 of them are included in the regression (to avoid redundancy). Next, for each residue in the target sequence, the hydrophobicity information of both the preceding and the succeeding residues are recorded. As a result, every residue, except the first and the last, is associated with one of the four labels: "HH", "HP", "PH", and "PP", where 'H' denotes hydrophobic and 'P' denotes hydrophilic. The frequency of "HH" is defined as the number of residues labeled with "HH" divided by *L *- 2. The other three frequencies are similarly defined, and their sum is exactly 1. For the same reason, only 3 of them are included in the regression.

We also tested two other regression methods: Support Vector Regression (SVR) [36] and Neural Network (NN) [37]. Combining these two regression methods, we have F3-SVR, F7-SVR, F27-SVR, and F3-NN, F7-NN, F27-NN. Performance of these nine different regression methods was assessed using a number of criteria to identify the best performing approach (see Results).

### Public web server

We have implemented the above contact order calculator, the homology-based contact order predictor, and the linear regression based contact order predictors as a public web server [38]. The input to the server can be either a three-dimensional structure (either uploading the PDB file or key in the PDB id), or the primary sequence of the query protein. When the input is a sequence, our server will first use BLAST to identify sequences that are either identical or homologous to those in our CO database. There are three possible scenarios: 1) If the input is a 3D structure, or the input sequence exactly matches a known structure in our database, our server will calculate its Abs_CO directly using Formula (1); 2) If the input is a sequence and the BLAST search finds a homolog that is not an exact match but satisfies the criteria described in the "Prediction by homology" section, the pre-computed Abs_CO of the homologue is used as the predicted Abs_CO of the query sequence; 3) If the input is a sequence and has no BLAST match that falls into the second scenario, our server will call Proteus to predict the secondary structure content for the query protein, and then report its Abs_CO using Formula (4). Average calculation times are around 35 seconds for the CO calculator and about 27 seconds for the CO predictor.

## Results and Discussion

**Figure 1:**
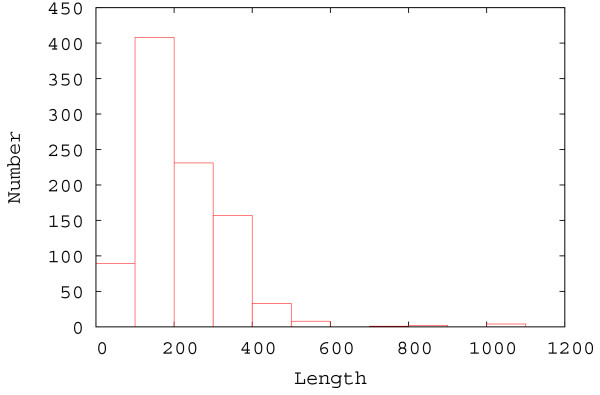
The length distribution of the 933 monomeric proteins used for regression.

All regression-based Abs_CO prediction methods were trained and tested on a set of 933 monomeric proteins with an X-ray resolution less than 1.5 Å, extracted from PDB [39]. The SCOP [40] classification and the length distribution of these 933 proteins are shown in Table 1 and Figure 1, respectively. Table 1 and Figure 1 show that these proteins come from a wide variety of structure categories and their lengths range from ~40 to ~1, 000 residues.

**Table 1:** SCOP classification of the 933 training monomeric proteins

SCOP label	Quantity
All alpha proteins	83
All beta proteins	89
Alpha and beta proteins	750
Peptides	10
unstructured	1

The SCOP classification of the 933 monomeric proteins used for regression.

### Contact order calculation

We implemented an absolute contact order calculator based on Formula (1) in the web server [38]. We tested our server using the monomeric protein dataset (described in the above "Prediction by homology" section) and compared it with a previously published contact order calculation server [30]. The two servers returned nearly identical contact order values with a correlation coefficient of 0.999. However, the other server failed (tested on May 2, 2007) to recognize 61% of the input PDB files while our server successfully processed all of the PDB files. As to the runtime, we observed no noticeable difference between the two servers.

### Results on homology-based prediction

**Figure 2:**
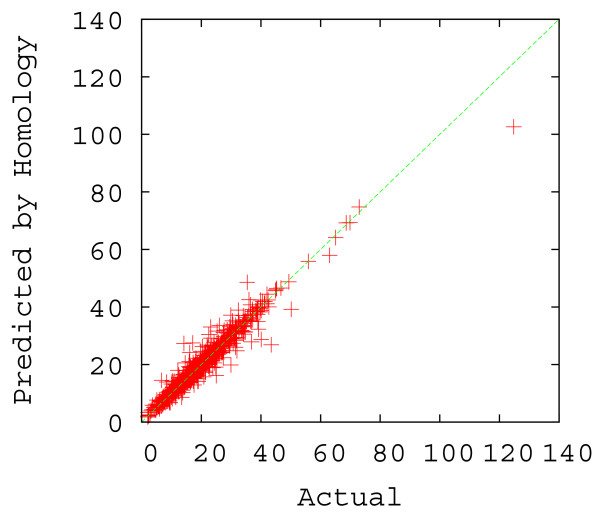
Scatter plot of the actual Abs_CO values of the 742 out of 1, 000 testing sequences versus the predicted Abs_CO values based on the top homology hit, where the 742 were obtained by setting the length difference threshold at 40% and the sequence identity threshold at 20%.

**Figure 3:**
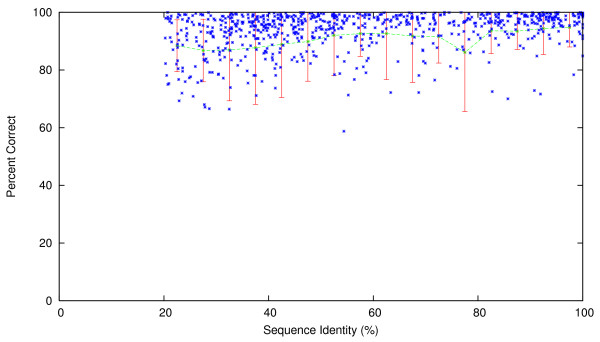
**Scatter plot for the sequence homology-based method, showing percent correct versus sequence identity for the 742 pairs of testing sequences and their corresponding homologs.** A 5-fold cross-validation was performed with 5 samples of 200 sequences each, and the combined results for all of the 742 sequences with homologs are plotted here. Sequence identity was computed as the number of identical residues divided by the query sequence length.

**Figure 4:**
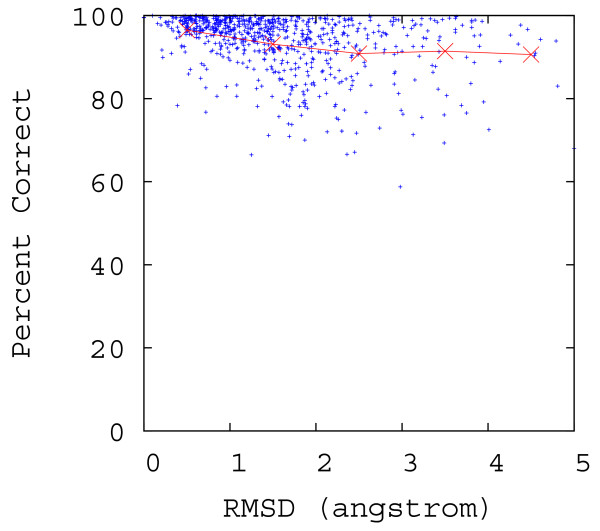
**Scatter plot for the sequence homology-based method, showing percent correct versus RMSD between the structure of the query protein and the structure of the homologous protein. **The experiment setting is the same as for generating Figure 3. In the plot, the red crosses show the average percent correct for their nearby points within ± 0.5 Å.

To test the accuracy of homology-based contact order prediction, a *modified *5-fold cross-validation was performed with a random sample of 1, 000 sequences. Each set of 200 out of the 1, 000 proteins was separately removed from the non-redundant sequence database to form the testing dataset, and each sequence in the testing dataset was queried against the remaining database (the training dataset) using BLAST. The known absolute contact order of each query was compared to the absolute contact order of the top-scoring BLAST hit. These top-scoring hits were subsequently filtered based on the 20% sequence identity threshold and 40% length threshold described above. On average 74.2% (± 2.2% standard deviation) of sample sequences found homologs in the database. The scatter plot of these 742 pairs of true absolute contact order and the predicted absolute contact order is shown in Figure 2, and the mean accuracy of these 742 predictions (measured as the average *percent correct*, defined for each prediction as min$\left\{\frac{a}{b},\frac{b}{a}\right\}$ where *a *is the true Abs_CO value of the query and *b *is the Abs_CO of the homolog), at intervals of 5% sequence identity, are plotted in Figure 3. Prediction of contact order by sequence homology turns out to be a surprisingly accurate prediction method, with a correlation coefficient of 0.977 between the 742 pairs of true absolute contact order and predicted absolute contact values. Abs_CO predictions are on average 93.4% correct (± 0.5% standard deviation), and are even strong at sequence identities as low as 20–30%, where they are on average over 86% correct (Figure 3). We also plot the percent correct versus the structure similarity, measured in RMSD, between the structure of the query protein and the structure of the homolog, in Figure 4, where we used CE [41], a protein structure comparison method, to compute the RMSD. From the figure, one can see that Abs_CO predictions are on average over 91% correct even when the structure similarity is larger than 3 Å. The 74 2% coverage we achieved with this test provides a good indication that CO prediction by homology can provide reliable answers for a significant number of queries. Obviously, a key limitation of a homology-based approach is that it cannot predict CO values if the query protein has no significant homologue. This is why the regression-based prediction is needed as a complement to the homology-based route.

### Results on regression-based prediction

In assessing the performance of our regression-based predictors we treated each protein in our monomeric protein dataset as a novel protein and applied each of the three linear regression based methods to predict its contact order. To train our contact order predictors, we extracted the primary sequences from the previously described database of 933 monomeric proteins, and used Proteus [31] to predict their secondary structure contents. The true Abs_CO value for each protein was calculated using its three-dimensional structure via Formula (1). We used a 5-fold cross validation scheme to avoid data over-fitting. We measured the prediction performance by calculating the correlation coefficient and the average prediction percent correct between the predicted Abs_CO values and the true Abs_CO values. The average percent correct is defined the same as in the last section (min$\left\{\frac{a}{b},\frac{b}{a}\right\}$ where *a *is the true value and *b *is the predicted value), averaged over all 933 proteins. Both the correlation coefficients and the average percent correct values for the three linear regression based prediction methods are listed in rows 3–5 in Table 2, Columns 2 and 5.

**Table 2:** Performances of all 9 regression-based Abs_CO prediction methods

	Correlation Coefficient			Average Percent Correct			Standard Deviation		
			
Method	LR	SVR	NN	LR	SVR	NN	LR	SVR	NN
Method 1 (F3)	0.8571	0.8583	0.8513	0.8630	0.8659	0.8543	0.1099	0.1060	0.1445
Method 1 (F7)	0.8603	0.8543	0.8737	0.8636	0.8681	0.8656	0.1091	0.1094	0.1110
Method 1 (F27)	0.8702	0.8658	0.7713	0.8659	0.8717	0.8086	0.1060	0.1291	1.2222
Method 2 (F3)	0.8477	0.8440	0.8680	0.8577	0.8664	0.8612	0.1179	0.1129	0.1073
Method 2 (F7)	0.8499	0.8455	0.8667	0.8570	0.8669	0.8656	0.1161	0.1142	0.1110
Method 2 (F27)	0.8662	0.8550	0.8233	0.8620	0.8714	0.7016	0.1105	0.1167	3.3827
Method 3 (F3)	0.8375	0.8378	0.8439	0.8542	0.8647	0.8560	0.1200	0.1168	0.1103
Method 3 (F7)	0.8421	0.8381	0.8636	0.8537	0.8637	0.8648	0.1192	0.1165	0.1156
Method 3 (F27)	0.8625	0.8512	0.7706	0.8605	0.8695	0.8106	0.1126	0.1194	0.3662

The correlation coefficients, the average percent correct values, and the standard deviations of the percent correct values for all 9 regression-based Abs_CO prediction methods. The first set of results (rows 3–5) are for Method 1 in which the percentage of residues in alpha helices, *p*(*α*), and beta strands, *p*(*β*), are used as two factors. In particular, the F3-LR regression formula is Abs_CO = -6.8968*p*(*α*) + 7.6216*p*(*β*) + 0.0612*L *+ 8.0397. The second set of results (rows 6–8) are for Method 2 in which the numbers of residues in alpha helices, *q*(*α*), and beta strands, *q*(*β*), are used as two factors. In particular, the F3-LR regression formula is Abs_CO = -0.0591*q*(*α*) + 0.0789*L *+ 8.3774. The third set of results (rows 9–11) are for Method 3 in which the numbers of alpha helices, *n*(*α*), and beta strands, *n*(*β*), are used as two factors. In particular, the F3-LR regression formula is Abs_CO = -0.4184*n*(*α*) + 0.1992*n*(*β*) + 0.0611*L *+ 8.647.

**Figure 5:**
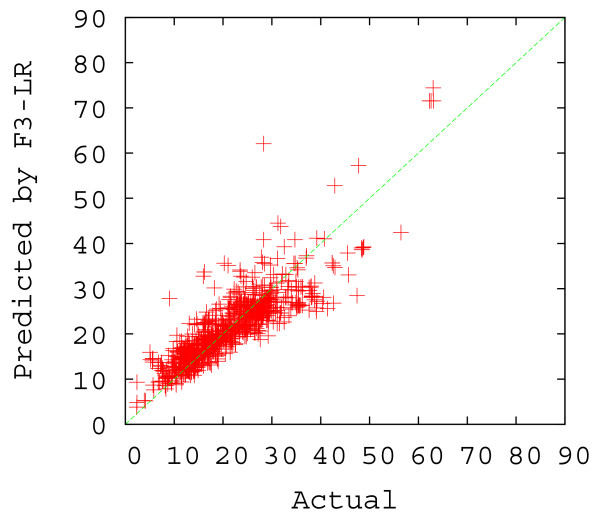
Scatter plot of the actual versus the predicted Abs_CO values by F3-LR (under the 5-fold cross validation scheme).

**Figure 6:**
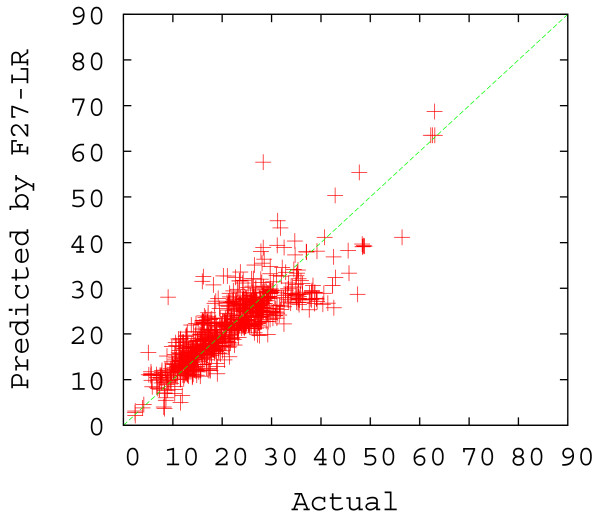
Scatter plot of the actual versus the predicted Abs_CO values by F27-LR (under the 5-fold cross validation scheme).

From Table 2 (rows 3–5), we see that while the use of more factors improves the correlation coefficient by a small amount, the simplest regression model (F3-LR) actually performed remarkably well. In fact, this 3-parameter approach yielded a correlation coefficient of 0.8571. Using 4 more factors, the F7-LR model improved the correlation coefficient by only 0.0032. Not unexpectedly, F27-LR had the best performance in terms of both correlation coefficient and average prediction percent correct. Due to the simplicity of the F3-LR model, it was chosen as the default method for our web server (the other two approaches are also available). The scatter plots of the predicted versus actual Abs_CO values using F3-LR and F27-LR are shown in Figures 5 and 6. As one can see from these plots, F27-LR slightly outperformed F3-LR, especially when the actual Abs_CO values are smaller than 10 or greater than 40.

Rows 6–11 in Table 2 summarize the correlation coefficients and the average percent correct values when the percentage of residues in alpha helices and beta strands (*p*(*α*) and *p*(*β*), Method 1) are substituted by the numbers of residues in alpha helices and beta strands (*q*(*α*) and *q*(*β*), Method 2), as well as by the numbers of alpha helices and beta strands (*n*(*α*) and *n*(*β*), Method 3), respectively, for all 9 regression based prediction methods. The three explicit three-factor linear regression formulae are also included in the caption. Two interesting observations are: 1) The coefficient for the term "number of residues in beta strands", *q*(*β*), is 0 in the second regression; 2) Using the percentage of residues in alpha helices and beta strands gave the best correlation coefficient, while using the other two sets of parameters on secondary structure content performed comparably well though slightly worse.

## Conclusion

In this paper, we proposed a simple yet very effective method to predict protein contact order from primary sequences. We discovered three factors (i.e., percentage of residues in alpha helices, percentage of residues in beta strands, and sequence length) that appear to be strongly correlated with the absolute contact order. Tests using a large dataset of high resolution monomeric proteins showed that our method achieved a correlation coefficient between the predicted and the actual absolute contact orders of 0.857–0.870. Several other factors were also identified and shown to correlate with the absolute contact order, including amino acid composition and adjacent residue hydrophobicity. In addition, we have also shown that it is possible to use sequence homology to accurately predict the contact order for proteins for which no 3D structure exists. This latter approach, which is extremely fast (less than a second) and accurate (correlation coefficient 0.977), avoids the need to have to generate and refine an accurate 3D homology model or to use extensive computer resources to calculate the contact order. Therefore, using a combination of homology-based prediction and regression-based prediction, we have shown that it is possible to rapidly and accurately predict the contact order of any water-soluble protein for which the sequence is known. All of these methods for CO prediction and calculation are freely available through the web server [38].

## Availability and requirements

Web server: .

## Authors' contributions

All authors participated in the prediction method design. YS, JZ and DA performed all the experiments, and all authors were involved in the result interpretation and discussion. YS, JZ, DA and GL drafted, and GL and DSW finalized the manuscript. All authors read and approved the final manuscript.
